# Technical note: Impact of beamline‐specific particle energy spectra on clinical plans in carbon ion beam therapy

**DOI:** 10.1002/mp.15652

**Published:** 2022-04-27

**Authors:** Andreas Franz Resch, Mansure Schafasand, Niklas Lackner, Tom Niessen, Staffan Beck, Alessio Elia, David Boersma, Loïc Grevillot, Piero Fossati, Lars Glimelius, Markus Stock, Dietmar Georg, Antonio Carlino

**Affiliations:** ^1^ Department of Radiation Oncology Medical University of Vienna Vienna Austria; ^2^ MedAustron Ion Therapy Centre Wiener Neustadt Austria; ^3^ RaySearch Laboratories AB Stockholm Sweden; ^4^ ACMITGmbH Wiener Neustadt Austria

**Keywords:** carbon ions, Monte Carlo simulations, RBE

## Abstract

**Purpose:**

The Local Effect Model version one (LEM I) is applied clinically across Europe to quantify the relative biological effectiveness (RBE) of carbon ion beams. It requires the full particle fluence spectrum differential in energy in each voxel as input parameter. Treatment planning systems (TPSs) use beamline‐specific look‐up tables generated with Monte Carlo (MC) codes. In this study, the changes in RBE weighted dose were quantified using different levels of details in the simulation or different MC codes.

**Methods:**

The particle fluence differential in energy was simulated with FLUKA and Geant4 at 500 depths in water in 1‐mm steps for 58 initial carbon ion energies (between 120.0 and 402.8 MeV/u). A dedicated beam model was applied, including the full description of the Nozzle using GATE‐RTionV1.0 (Geant4.10.03p03). In addition, two tables generated with FLUKA were compared. The starting points of the FLUKA simulations were phase space (PhS) files from, firstly, the Geant4 nozzle simulations, and secondly, a clinical beam model where an analytic approach was used to mimic the beamline. Treatment plans (TPs) were generated with RayStation 8B (RaySearch Laboratories AB, Sweden) for cubic targets in water and 10 clinical patient cases using the clinical beam model. Subsequently, the RBE weighted dose was re‐computed using the two other fluence tables (FLUKA PhS or Geant4).

**Results:**

The fluence spectra of the primary and secondary particles simulated with Geant4 and FLUKA generally agreed well for the primary particles. Differences were mainly observed for the secondary particles. Interchanging the two energy spectra (FLUKA vs. GEANT4) to calculate the RBE weighted dose distributions resulted in average deviations of less than 1% in the entrance up to the end of the target region, with a maximum local deviation at the distal edge of the target. In the fragment tail, larger discrepancies of up to 5% on average were found for deep‐seated targets. The patient and water phantom cases demonstrated similar results.

**Conclusion:**

RBE weighted doses agreed well within all tested setups, confirming the clinical beam model provided by the TPS vendor. Furthermore, the results showed that the open source and generally available MC code Geant4 (in particular using GATE or GATE‐RTion) can also be used to generate basic beam data required for RBE calculation in carbon ion therapy.

## 1 INTRODUCTION

The response of biological tissue to ionizing radiation is complex and depends on a variety of biological and physical parameters. The absorbed dose is in most cases a sufficient single parameter descriptor of the radiation field in high energy photon beam therapy. However, in carbon ion beam therapy, an additional parameter, the relative biological effectiveness ($RBE\;=\;\frac{D_{ref}}{D_{abs}}{|}_{iso-effect}$, is required such that an absorbed carbon dose (*D_abs_*) of some beam quality yields the same biological effect as at a dose level (*D_ref_*) with reference beam quality. In addition to the radiation quality, RBE depends on multiple factors such as the absorbed dose level and the biological properties of the tissue. Due to the complexity in modeling the tissue‐specific biological properties (tissue sensitivity, immunological sensitivity, and others), RBE models and predictions are subject of debate and intensive research. RBE typically ranges in the order of about 1.5 to 3 for a typical clinical dose range,^1^ but strongly depends on the endpoint and the tissue sensitivity and is accompanied with uncertainties in the order of 20–30%.^2^, ^3^, ^4^, ^5^ As absorbed dose to water can be determined with ionization chambers up to an accuracy of 3% for carbon ions,^6^ RBE is the major source of uncertainty in clinical application of carbon ions.

Although numerous RBE models are reported in literature, only three have been applied clinically: the local effect model I (LEM I),^7^ a semiempirical model proposed by Kanai et al.,^8^ and the modified microdosimetric kinetic model (mMKM).^9^ All RBE models require a descriptor of the beam quality such as the local particle fluence differential in energy as input parameter to calculate the RBE. Pre‐calculated and beamline specific look‐up tables are typically used in treatment planning systems (TPSs) to allow calculation times feasible for daily clinical work. The tabulated particle fluence as a function of depth and initial pencil beam (PB) energy are typically created with general purpose Monte Carlo (MC) particle transport simulation codes such as FLUKA or Geant4.^10^, ^11^, ^12^, ^13^ Although such MC codes are computationally intensive, they are currently the most accurate option to calculate the particle transport in matter. For carbon ion beams, nuclear interactions of primary carbon ions with nuclei in the beamline or the phantom/patient remove a substantial fraction of the primary particles and create—mostly lighter—secondary fragments, which finally result in a mixed particle spectrum. A clinical carbon beamline contains vacuum windows, beam monitoring devices, optional range shifters, and ripple filters. The center‐specific position and material composition of those devices influences the beam, which needs to be accounted for in the beam model. Consequently, the spectra depend on the clinic as well as the approach of TPS vendors.

The purpose of the current study was to quantify the clinical impact on RBE weighted dose of creating those fluence look‐up tables with different levels of details in the simulation and different MC codes.

## 2 MATERIALS AND METHODS

### 2.1 Overview

Three beam models were created in the treatment planning system RayStation version 8B SP1 (RaySearch Laboratories AB, Stockholm, Sweden) for a horizontal carbon beamline using differently generated particle fluence look‐up tables. Note that this will only affect the computation of the RBE weighted dose; the absorbed dose computation is based on beam model data, which is common for the three options. The impact on the RBE was then systematically investigated in regular shaped targets of different sizes and depths in water, and the clinical impact was investigated for 10 patient cases. This was an in silico study, and no comparison to experiments was carried out.

Two simulations included the exact geometric description of the Nozzle, one using Geant4 and one using FLUKA in the following referred to as FLUKA PhS, as it uses the Geant4 phase space files at Nozzle exit as source. Using the GATE/Geant4‐generated phase space has the advantage that the beam at Nozzle exit is exactly the same allowing for a direct comparison between the MC codes eliminating inevitable differences coming from the beam modeling process. The third setup is the clinical beam model based on FLUKA simulations, but using an analytical description of the Nozzle contributions. An overview of the three simulation setups can be found in Figure S1.

### 2.2 Simulations to generate particle fluence tables

Particle energy spectra were obtained for quasimonoenergetic carbon ion beams in water with initial energies ranging from 50 to 450 MeV/u in 5 MeV/u steps. In all MC simulations, track‐weighted fluence was scored using the ratio of the sum of track lengths within a region divided by its volume.^14^ This was found to be more reliable than the planar approximation, where a particle entering a plane is weighted by the inverse of the cosine of its incoming angle, which is unstable at angles close to ±π/2.

The scoring volume was a cylinder with radius of 5 cm placed within a 50 × 50 × 50 cm^3^ water phantom oriented with its axial axis along beam direction. The fluence differential distributions in energy of all particles with charge +1 to +6 were scored per ion species in slabs of 1 mm thickness oriented perpendicular to the beam direction. The fluence of primary particles was scored in 550 energy bins uniformly distributed from 0 to 550 MeV/u, while a logarithmic energy binning was applied for secondary particles using 133 bins from 0.1 to 990 MeV/u. The latter lower limit of the energy binning was chosen in accordance with results from other work.^15^ Fluence was scored with a 1 mm resolution in depth resulting in 500 times 6 fluence spectra per initial beam energy. A general overview of the MC settings is given in the following, and technical details are listed in the Supporting Information.

#### 2.2.1 GATE/Geant4

Geant4.10.3.p02 simulations were carried out using a custom patch of GateRTion V1.0,^16^, ^17^ with an additional option in the EnergySpectrumActor enabling to extract the particle fluence. We implemented and validated the fluence scoring option in Gate 8.2, where it is available since version 8.2 and may be available in a future release of GateRTion.

Particles were tracked through a detailed model of the MedAustron Nozzle, which was previously developed and extensively validated for proton beams.^18^, ^19^

Two sets of simulations were performed: firstly, the fluence tables of all particles at all depths in water were directly created for all energies, and secondly, a phase space (including the information of the particle type, momentum, and position) was created at the Nozzle exit window.

#### 2.2.2 FLUKA

The phase space at the Nozzle exit window was used as a starting point for one of the two FLUKA simulations. In this way, the Nozzle elements were explicitly simulated in the FLUKA PhS setup, whereas an analytical approximation was used for the clinical beam model.

For the clinical model, monoenergetic particle energy spectra kernels were simulated and stored as RayStation base data. In order to compute a beamline specific spectrum, the kernels are convolved with the initial energy spectra of the spot. An analytical model of the nozzle was then applied by offsetting the convolved spectrum with a distribution of water equivalent thicknesses of the ripple filter and other nozzle‐specific materials. The final spectrum was computed as a weighted sum, where the weights were obtained by fitting the corresponding laterally integrated dose distributions to measured depth dose curves.

### 2.3 Treatment plans and analysis

All treatment plans (TPs) were created applying the clinical beam model and subsequently recomputed within RayStation 8B SP1 applying the two other beam models.

The analysis of the impact of the fluence spectra for RBE was divided into three groups:

1. Investigation of the *input tables* (particle fluences). To investigate the differences in the energy spectrum as a function of depth, the particle spectra were integrated over the energy at each depth. The spectra were split into a low and high energy region with an energy threshold corresponding to 2 mm continuously slowing down approximated range in water. The energy threshold was derived from SRIM2013 for each particle. The 2 mm range threshold was chosen, as it refers to end of range effects and voxel sizes in a TPS are typically in that order.
2. Consequences for RBE weighted dose in *cubic shaped targets* and a single field. The plans were optimized to yield an RBE weighted dose equal to 2.3 Gy(RBE), which is a lower dose than typically applied in clinical plans and makes the RBE differences more visible. Three cubic targets were created with 6, 8, and 10 cm side length centered at 6, 8, and 21.8 cm depth, respectively. Those boxes are referred to in the following by side length and position in depth, e.g., for the 10 cm side length target located at 21.8 cm “box 10/21.8.”
3. Re‐calculation of *10 patient cases* irradiated with carbon ions, i.e., six head and neck, three pelvis, and one THORAX patient. Clinical parameters were investigated to characterize the dose distribution in terms of RBE weighted dose volume histograms (DVHs). D_98%_, D_50%_, and D_2%_ were evaluated for planning target volumes (PTVs). The parameters for OARs encompassed the mean RBE weighted dose, D_20%_, D_10%_, D_5%_, D_2%_, D_1%_, and D_0.01%_. A uniform *α*/*β* ratio of 2 Gy was applied in the LEMI as is used clinically.

## 3 RESULTS

### 3.1 Comparison of fluence distributions

Two datasets generated with FLUKA PhS and Geant4 were qualitatively in good agreement, particularly for the primary particles, which contribute most to the dose deposition. Notable deviations appeared for lithium and beryllium (atomic numbers 3 and 4). However, the fluence of those particles with highest deviations was about one order of magnitude lower than the fluence of secondary protons and helium ions, respectively. Two examples of particle fluence distributions in the entrance plateau and at the Bragg peak of a high energy carbon ion beam (400.0 MeV/u) can be found in Figure S2.

The fluence of the primary ions reduces over depth to less than half of the initial value for the high energy in both data tables, where GATE/Geant4 predicted slightly less removal of primary ions than FLUKA PhS. Up to roughly half range, secondary particle fluences agreed well between the two codes, but deviations increased at larger depths. Two major deviations were evident: GATE/Geant4 predicted more protons and less helium ions compared to FLUKA PhS. In Figure S3a,b, one can find the primary and secondary particle fluence integrated over the entire energy range as a function of depth.

GATE/Geant4 generally yielded less low energy secondary protons and helium ions compared to FLUKA PhS. However, the absolute difference was small, as the low energy abundance was low. GATE/Geant4 yielded more high energy protons and less high energy helium ions compared to FLUKA PhS. Consequently, the fluence differences were mainly caused by the differences in the high energy regime.

### 3.2 Regular shaped targets in water

**FIGURE 1:**
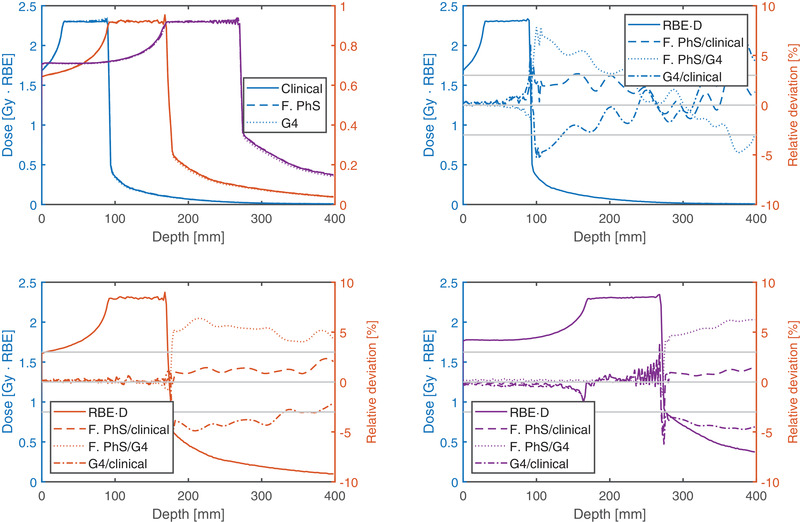
RBE weighted dose profiles along the central beam axis of the three cubic‐shaped targets are plotted in the top left panel and the relative deviations in the remaining panels. The box 6/6 (side length/depth), box 8/13, and box 10/21.8 can be identified by the colors blue, red and purple, respectively. Gray horizontal lines indicate the ±3% deviation level. The results of the clinical, the FLUKA PhS, and GATE/Geant4 beam models are shown

The RBE weighted dose profiles along the central axis resulting from the three beam models in the three cubic‐shaped targets in water are shown in Figure 1. The corresponding dose volume histogram (DVH) parameters for the entrance, target, and fragmentation tail region are listed in Table S1. There were no relevant systematic differences in the entrance up to the proximal target region with one exception. For the deepest box about 4 mm before the SOBP a 2% deviation between the clinical beam model and the two beam models with an explicit description of the nozzle could be observed.

In the target region without the distal 2 mm, the two beam models created with the detailed Nozzle description (FLUKA PhS and G4) resulted in less than 0.5% higher RBE than the clinical beam model. In the distal 2 mm, the RBE was up to 3 and 2% higher than the GATE/Geant4 and FLUKA PhS with respect to the clinical beam model. This was more pronounced at the shallowest target and vanished for the deepest target.

In the fragmentation tail, the RBE was locally up to 2–4% higher and 5% lower using the tables generated with FLUKA PhS and GATE/Geant4 compared to FLUKA clinical, respectively. While the deviations of the FLUKA PhS (against the clinical beam model) tended to decrease with the depth of the target, the deviations of the GATE/Geant4 based RBE calculations increased with depth. This trend was also observed in the D_98%_ and D_50%_ parameters in the volume encompassing the fragmentation tail (see Table S1).

### 3.3 Clinical treatment plans

**FIGURE 2:**
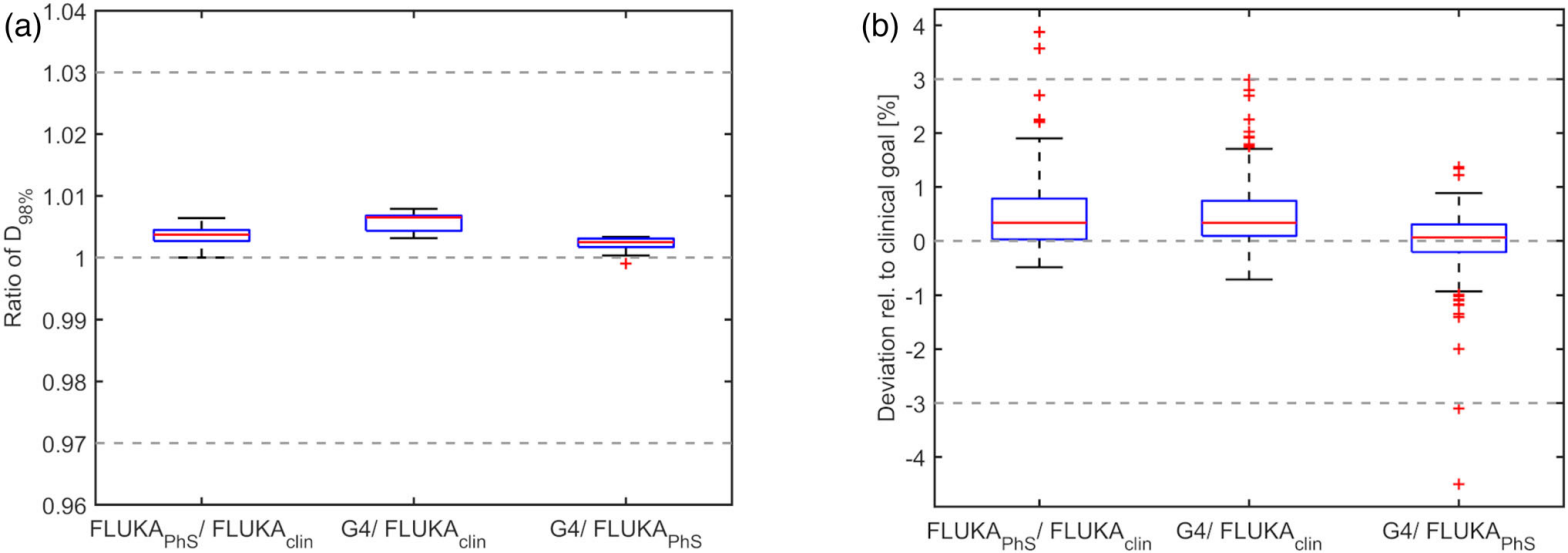
Influence of the particle spectra on DVH parameters of all patient cases represented with boxplots. The ratio of the D_98%_ parameters of the PTV is shown in the left panel. The difference of all 199 evaluated DVH parameters of the OARs is shown on the right relative to the clinical goal. The red line in the box represents the median, the upper, and lower edges of the box the 25^th^ and 75^th^ percentiles; the whiskers the most maximum and minimum (following Tukey definition) excluding outliers which are represented with red + symbols

The RBE weighted dose volume histograms of two exemplary patients can be found in Figure S4. In both examples, the nominal beam model resulted in lower PTV doses. A boxplot of the relative ratio of the D_98%_ is shown in Figure 2a. The RBE weighted dose was higher when using FLUKA PhS or GATE/Geant4, but the deviation with less than 1% was negligible.

In the two exemplary patient DVHs, larger deviations become apparent in the CTVs. The D_2%_ in the brainstem of patient case shown in Figure S4a was 44.8 Gy(RBE) and was 0.2 Gy(RBE) higher when recomputed with the two other beam models, but still being below the clinical goal of 50 Gy(RBE). In the second example, one of the two clinical constraints on the nerve roots was reached with the clinical beam model, while the recalculation resulted in 0.3 and 0.5 Gy(RBE) higher values, which was about 0.4 and 0.6% relative to the clinical constraint. To account for the different organ sensitivity and the different dose levels received, the OAR DVH parameter deviation of the patient population was calculated relative to the individual clinical goal. The observation of higher doses in the two recomputed scenarios also holds true for the median of all 199 evaluated DVH parameters, but with a relative deviation of less than 0.5% being clinically not relevant.

## 4 DISCUSSION

Our study demonstrated that primary and secondary particle fluence distributions were sensitive to underlying MC codes or methods to account for the nozzle influence. Yet, different particle spectra did not alter the computed RBE values in the target region more than 1%. RBE was solely altered in the distal layer of a target located at a shallow depth and in the fragmentation tail of a monodirectional beam. Multidirectional beams, which are typically applied for patient treatment, wash out the systematic deviations of a single beam. Consequently, the observed DVH parameter deviations in the patient cohort investigated in this study were minor. Deviations in the OARs were small and considered to not change the clinical decision. The few cases with higher than 3% deviations originated in discretization artifacts, where the finite voxel size in small volumes caused an increase of the deviation, which amplified the numerical deviation. The impact of uncertainties in particle fluence stemming from MC and Nozzle modeling on RBE weighted doses were much smaller than RBE inherent uncertainties.^1^, ^2^, ^3^, ^20^ The low sensitivity of RBE on fragment spectra found in this study is consistent with literature.^21^

RBE values in the fragmentation tail were lower when using GATE/Geant4 compared to FLUKA, which can be explained by the differences in the particle fluences. GATE/Geant4 yielded more protons and less helium ions, resulting in a lower RBE since helium ions have a higher RBE compared to protons. The differences in the proton and helium yield and the RBE in the fragmentation tail increased with increasing energy. Local deviations were shown in the monodirectional beam arrangement. The global deviations (with respect to target dose) are at least a factor of 2.7 smaller than the local deviations, and this factor increases with depth and with decreasing initial energy.

The TPS version used in this study as well as syngo PT Planning (Siemens, Germany) and TRiP98^22^ use an infinite‐slab approximation for fluence and LEM I‐based RBE calculation. Physically, this approximation assumes that the nuclear scattering cross‐sections have no angular dependence. However, light secondary fragments scatter under larger angles compared to heavier secondaries, which makes the central Gaussian part of a single pencil beam dominated by heavier fragments (and the primaries), whereas the outer part is vastly dominated by protons.^23^ Consequently, there is a RBE variation with lateral position, which has been experimentally verified in the fragmentation tail.^24^ The lateral RBE variation reported by Hirano et al. was about one order of magnitude higher than the uncertainties observed in our study. Hence, future studies may need to emphasize the lateral nonuniform RBE and how to translate current clinical experience to a more sophisticated calculation method.

In the TPS version used in this study, only LEM I was available for clinical RBE calculation. Newer versions of the TPS offer the possibility to use the mMKM. How the results of this LEM I‐based study translate to the mMKM remains to be demonstrated, but similar results may be expected since the RBE and dose are dominated by the primary particles. Good agreement of the primary particle fluence was observed, which is consistent with literature reporting that total charge changing cross‐sections are generally well modeled in both MC codes.^25^

## 5 CONCLUSIONS

The influence of the secondary particle tables on LEM I‐based RBE weighted dose computation were clinically not relevant. As RBE weighted dose in OARs was most sensitive to the different ways of particle spectra table generation, such deviations may be considered for retrospective normal tissue complication analysis.

Both Geant4 (in particular using GATE or GATE‐RTion) and the analytical description of the Nozzle in the TPS can be used to generate beamline specific basic beam data required for RBE calculation in carbon ion therapy.

## CONFLICT OF INTEREST

Some authors are affiliated with the vendor of the TPS used in this study. This had no influence on the outcome or design of the study.

## Supporting information



Supporting Information

Supporting Information

Supporting Information

Supporting Information

Supporting Information

Supporting Information

Supporting Information
