# Supplementary material for: Technical note: Impact of beamline‐specific particle energy spectra on clinical plans in carbon ion beam therapy
Source: Med Phys. 2022 Apr 27;49(6):4092–8. doi: 10.1002/mp.15652 (PMC9321194; doi:10.1002/mp.15652)
Supplement: Supplementary file 6 — Supporting Information [file MP-49-4092-s003.pdf]

Supplementary materials to:

Impact of beamline-specific particle energy spectra on clinical plans in carbon ion beam therapy

Andreas Franz Resch[2], Mansure Schafasand[1], Niklas Lackner[1], Tom Niessen[3], Staffan Beck[3], Alessio Elia[1], David Boersma[1,5], Loïc Grevillot[1], Piero Fossati[1], Lars Glimelius[3], Markus Stock[1], Dietmar Georg[2], Antonio Carlino[1]

[1] MedAustron Ion Therapy Centre, Marie-Curie-Strasse 5, Wiener Neustadt, Austria

[2] Medical University of Vienna, Department of Radiation Oncology, Waehringer Guertel 18-20, 1090 Vienna, Austria

[3] RaySearch Laboratories AB, Eugeniavägen 18, PO Box 3297, Stockholm, Sweden

[5] ACMITGmbh, 2700 Wiener Neustadt, Austria

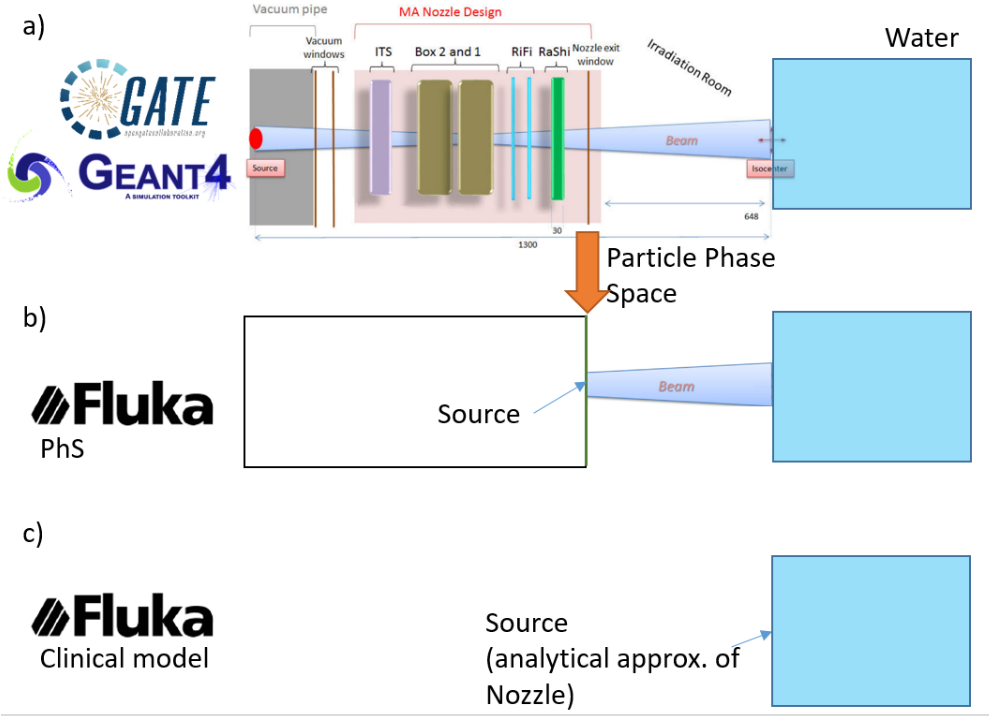

Figure 1 Overview of the three simulation setups with increasing detailedness of the geometry from bottom to top. The beam is coming from the left, passing through the Nozzle and entering the water phantom at the room isocenter after 65 cm of air gap. The nozzle specifications in a) are re-drawn from Stock et al.[16] with permission from the publisher.

1. Simulation Details

a. Geant4

To generate the beam model, beam parameters (initial energy distribution, beam width, divergence and emittance) were altered such that they reproduced dose profiles measured in air at several distances from the isocenter and laterally integrated dose distributions as a function of depth (IDDs) measured in a water phantom placed at isocenter [19]. The quantum molecular dynamics model (QMD) was activated for nuclear interactions of GenericIons and the binary cascade for protons and neutrons activated via the physics list "Shielding". Electromagnetic interactions were modeled using option 4 (EMZ). The maximum step limit was set to 0.1 mm and production cut for secondary protons to 0.01 mm, whereas the production of e-, e+ and gamma was inhibited by using a high production cut. In each run 10^7 events were simulated. The target material was G4_WATER with a mean excitation potential of 78 eV.

b. Fluka

FLUKA2011 was used in all simulations with the recommended default settings for hadrontherapy. This included heavy ion nuclear interactions and production of delta rays above a kinetic energy of 100 keV. In addition, the maximum step size for the transport of hadrons and electrons was overwritten to 0.15 cm in order to decrease statistical fluctuations (setting STEPSIZE). The user routine *mgdraw* was used to output the relevant phase space of each energy loss event in the simulation.

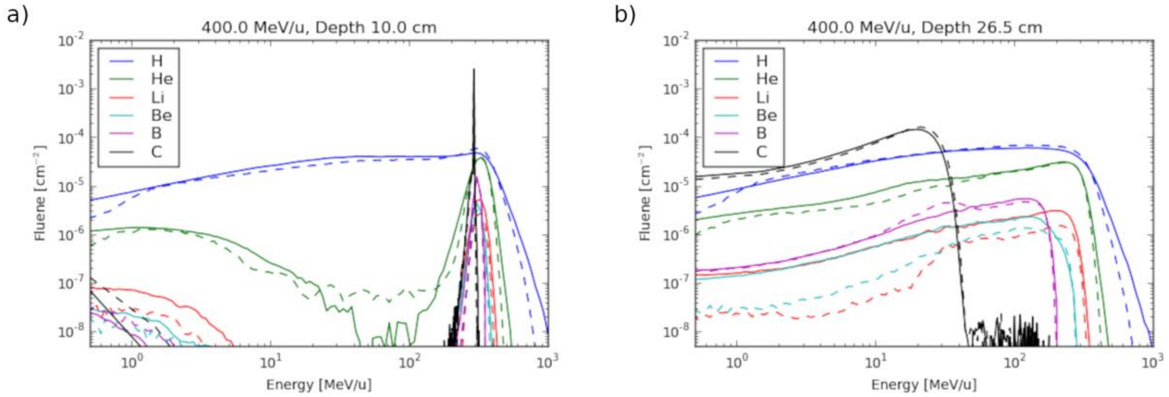

Figure 2 The particle fluence differential in energy at a constant depth in the entrance plateau (left) and the Bragg peak (right) in water of a primary carbon ion beam with 400 MeV/u initial energy. FLUKA PhS and GATE/Geant4 fluences are plotted with solid and dashed lines, respectively.

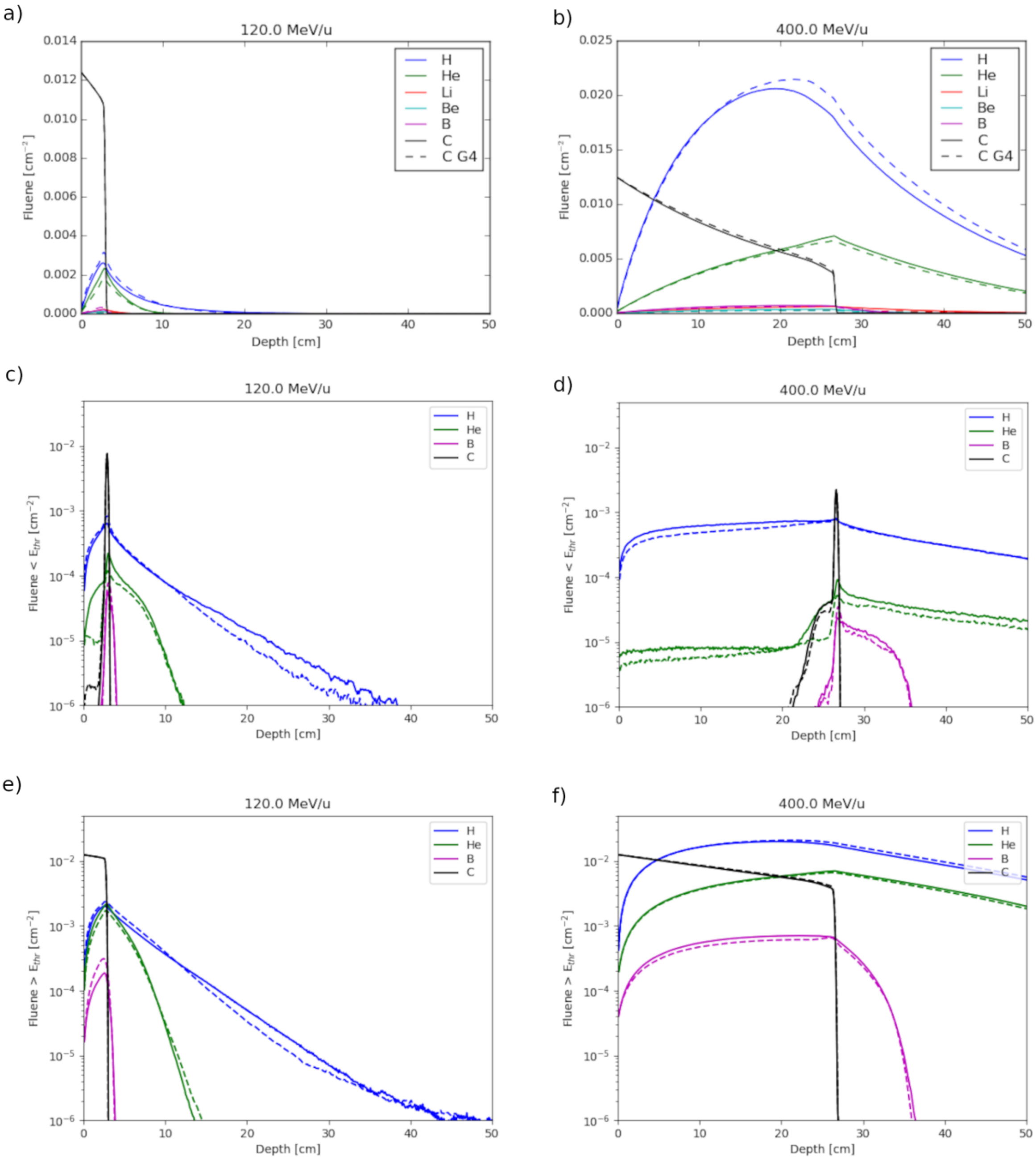

Figure 3 Fluence over depth of a 120 (left) and 400 MeV/u (right) carbon ion beam in water. Total fluence is shown in the first row. In the second and third row fluence was split into the low (middle row) and high energy (bottom row) contributions by applying an energy threshold, E_{thr}, corresponding to 2 mm range in water. The secondary and primary particles can be identified with different colors, while the line shape represents the different simulation setup; solid lines show FLUKA PhS and dashed lines show GATE/Geant4 particle fluences.

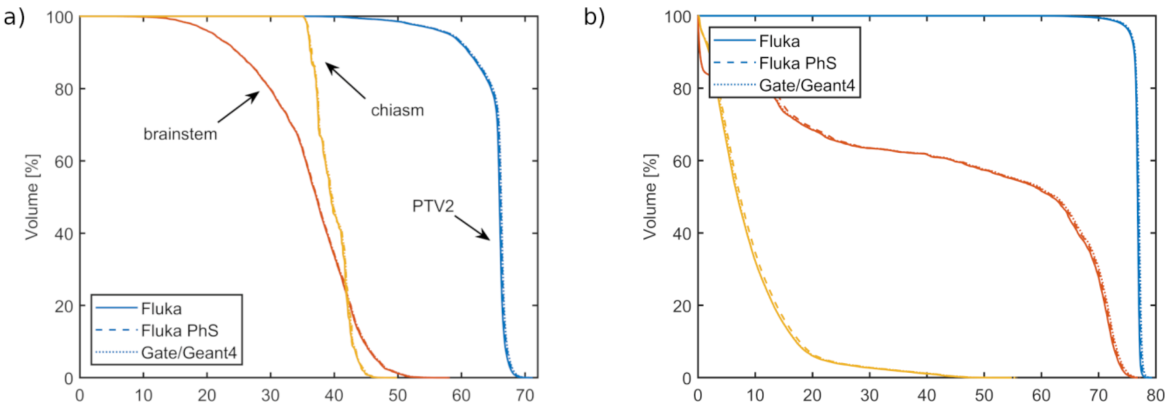

Figure 4 Influence of the particle spectra on the DVHs of one head (left) and one pelvis (right) patient.The PTV is represented with blue colors and the OARs with red (brainstem, nerveroots) and yellow (chiasm, rectum), respectively

Table 1 Relative dose deviation in the entrance, target and fragmentation tail region of three treatment plans in water. The three TPs were optimized to have a uniform dose of 2 Gy(RBE) in cubes with side length 6, 8 and 10 cm centered at 6, 13 and 21.8 cm depth, respectively. The entrance region was defined until as the region until begin of the target and the fragmentation tail from 2mm behind the target until 5 cm. The median dose (D50), the near maximum dose (D0.01) and D98% are given in % deviation. PhS and clinical represent the results of the beam model created with FLUKA using the phase space and clinical approach, respectively. G4 refers to the GATE/Geant4 results.

	D98%			D50%			D0.01%		
	G4/PhS	PhS/clin	G4/clin	G4/PhS	PhS/clin	G4/clin	G4/PhS	PhS/clin	G4/clin
**Entrance**									
Box 6/6	0.0	0.0	0.0	0.0	0.5	0.4	0.0	0.7	0.7
Box 8/13	0.0	0.0	0.0	-0.1	0.0	0.0	0.0	-0.4	-0.3
Box 10/21.8	-0.2	-0.1	-0.3	-0.2	-0.1	-0.3	-0.1	0.2	0.2
**Target**									
Box 6/6	0.1	0.5	0.7	0.1	0.4	0.5	1.2	0.7	1.9
Box 8/13	0.6	0.1	0.7	0.1	0.1	0.1	0.2	0.0	0.2
Box 10/21.8	-0.1	1.2	1.1	-0.1	0.4	0.3	0.3	-0.6	-0.3
**Fragmentation tail**									
Box 6/6	-3.6	2.0	-1.7	-5.2	2.5	-2.8	1.2	1.8	3.0
Box 8/13	-5.3	1.4	-4.0	-5.7	1.1	-4.7	0.4	0.9	1.3
Box 10/21.8	-5.9	0.7	-5.2	-5.3	0.8	-4.5	0.4	-2.4	-1.9
