# Supplementary material for: Technical note: Impact of beamline‐specific particle energy spectra on clinical plans in carbon ion beam therapy
Source: Med Phys. 2022 Apr 27;49(6):4092–8. doi: 10.1002/mp.15652 (PMC9321194; doi:10.1002/mp.15652)
Supplement: Supplementary file 7 — Supporting Information [file MP-49-4092-s001.rtf]

	D98%	D50%	D0.01%	
	G4/PhS	PhS/clin	G4/clin	G4/PhS	PhS/clin	G4/clin	G4/PhS	PhS/clin	G4/clin	
Entrance
Box 6/6	0.0	0.0	0.0	0.0	0.5	0.4	0.0	0.7	0.7	
Box 8/13	0.0	0.0	0.0	-0.1	0.0	0.0	0.0	-0.4	-0.3	
Box 10/21.8	-0.2	-0.1	-0.3	-0.2	-0.1	-0.3	-0.1	0.2	0.2	
Target	 		 	 		 	 			
Box 6/6	0.1	0.5	0.7	0.1	0.4	0.5	1.2	0.7	1.9	
Box 8/13	0.6	0.1	0.7	0.1	0.1	0.1	0.2	0.0	0.2	
Box 10/21.8	-0.1	1.2	1.1	-0.1	0.4	0.3	0.3	-0.6	-0.3	
Fragmentation tail	 		 	 		 	 			
Box 6/6	-3.6	2.0	-1.7	-5.2	2.5	-2.8	1.2	1.8	3.0	
Box 8/13	-5.3	1.4	-4.0	-5.7	1.1	-4.7	0.4	0.9	1.3	
Box 10/21.8	-5.9	0.7	-5.2	-5.3	0.8	-4.5	0.4	-2.4	-1.9	
